# Radiotherapy dose escalation using pre-treatment diffusion-weighted imaging in locally advanced rectal cancer: a planning study

**DOI:** 10.1093/bjro/tzad001

**Published:** 2023-12-12

**Authors:** Nathan Hearn, Alexandria Leppien, Patrick O’Connor, Katelyn Cahill, Daisy Atwell, Dinesh Vignarajah, Myo Min

**Affiliations:** Department of Medical Imaging, Sunshine Coast University Hospital, Birtinya, QLD 4575, Australia; Thompson Institute, University of the Sunshine Coast, Birtinya, QLD 4575, Australia; Department of Radiation Oncology, Sunshine Coast University Hospital, Birtinya, QLD 4575, Australia; Department of Radiation Oncology, Sunshine Coast University Hospital, Birtinya, QLD 4575, Australia; School of Information Technology and Electrical Engineering, University of Queensland, St Lucia, QLD 4072, Australia; Thompson Institute, University of the Sunshine Coast, Birtinya, QLD 4575, Australia; Department of Radiation Oncology, Sunshine Coast University Hospital, Birtinya, QLD 4575, Australia; Thompson Institute, University of the Sunshine Coast, Birtinya, QLD 4575, Australia; Department of Radiation Oncology, Sunshine Coast University Hospital, Birtinya, QLD 4575, Australia; Department of Radiation Oncology, Sunshine Coast University Hospital, Birtinya, QLD 4575, Australia; School of Medicine and Dentistry, Griffith University, Sunshine Coast Health Institute, Birtinya, QLD 4575, Australia; Thompson Institute, University of the Sunshine Coast, Birtinya, QLD 4575, Australia; Department of Radiation Oncology, Sunshine Coast University Hospital, Birtinya, QLD 4575, Australia; School of Medicine and Dentistry, Griffith University, Sunshine Coast Health Institute, Birtinya, QLD 4575, Australia

**Keywords:** Rectal cancer, Radiotherapy, Dose escalation, Diffusion-weighted imaging, MRI

## Abstract

**Objectives:**

Diffusion-weighted MRI (DWI) may provide biologically relevant target volumes for dose-escalated radiotherapy in locally advanced rectal cancer (LARC). This planning study assessed the dosimetric feasibility of delivering hypofractionated boost treatment to intra-tumoural regions of restricted diffusion prior to conventional long-course radiotherapy.

**Methods:**

Ten patients previously treated with curative-intent standard long-course radiotherapy (50 Gy/25#) were re-planned. Boost target volumes (*BTVs*) were delineated semi-automatically using 40th centile intra-tumoural apparent diffusion coefficient value with expansions (anteroposterior 11 mm, transverse 7 mm, craniocaudal 13 mm). Biased-dosed combined plans consisted of a single-fraction volumetric modulated arc therapy flattening-filter-free (VMAT-FFF) boost (phase 1) of 5, 7, or 10 Gy before long-course VMAT (phase 2). Phase 1 plans were assessed with reference to stereotactic conformality and deliverability measures. Combined plans were evaluated with reference to standard long-course therapy dose constraints.

**Results:**

Phase 1 BTV dose targets at 5/7/10 Gy were met in all instances. Conformality constraints were met with only 1 minor violation at 5 and 7 Gy. All phase 1 and combined phase 1 + 2 plans passed patient-specific quality assurance. Combined phase 1 + 2 plans generally met organ-at-risk dose constraints. Exceptions included high-dose spillage to bladder and large bowel, predominantly in cases where previously administered, clinically acceptable non-boosted plans also could not meet constraints.

**Conclusions:**

Targeted upfront LARC radiotherapy dose escalation to DWI-defined is feasible with appropriate patient selection and preparation.

**Advances in knowledge:**

This is the first study to evaluate the feasibility of DWI-targeted upfront radiotherapy boost in LARC. This work will inform an upcoming clinical feasibility study.

## Introduction

Locally advanced rectal cancer (LARC), including locally invasive T3 or T4 disease, is typically treated with neoadjuvant long-course chemoradiotherapy (NCRT) consisting of 45-50.4 Gy/25-28# with concurrent fluoropyrimidine chemotherapy, followed by surgical resection. At time of surgery, pathologic complete response (pCR) is found in only approximately 15% of patients.^1^ Improving locoregional control with neoadjuvant therapy may reduce surgical morbidity and allow for surgery-sparing or “wait-and-watch” approaches.^2^

Meta-analyses have identified higher rates of pCR and histopathological major response with dose escalation to >60 Gy^3^ or >54 Gy with modern inverse-planning techniques,^4^ and earlier trials hypothesized a steep dose–response relationship in local disease regression above 50 Gy.^5^ More recently, the RECTAL-BOOST randomized controlled study found increased rates of major histopathological response with sequential 15 Gy/5# + 50 Gy/25# radiotherapy, but no difference in pCR^6^ and transient quality of life detriments.^7^

Targeted dose intensification may play a role in ameliorating the compromise between locoregional control and toxicity. Functional imaging sequences such as diffusion-weighted MRI (DWI) can identify theoretically radioresistant tumours and can guide targeted dose escalation.^8^^,^^9^ While a prior trial utilizing PET-CT to boost more metabolically active tumour areas to up to 60 Gy did not identify any benefit in doing so,^10^ DWI may be a superior modality for functional delineation. It is routinely acquired pre-treatment for local staging and can be used to identify functional subvolumes within anatomically accurate gross tumour contours based on co-registered T2-weighted sequences.^11^ This may yield more specific tumour volumes: A recent comparison of rectal tumour contours between imaging modalities described smaller treatment volumes with DWI compared with PET-CT.^12^

Highly conformal dose delivery is facilitated by the use of inverse-planning techniques such as volumetric modulated arc therapy (VMAT). Flattening-filter-free VMAT (VMAT-FFF) has been used for its favourable dosimetric properties in reducing scatter to non-target tissue (NTT) and shorter delivery times, typically in the context of stereotactic body radiation therapy (SBRT) and other hypofractionated therapies.^13^ Ostensibly, these properties may suit the delivery of targeted dose escalation in rectal cancer radiotherapy, given the potential for intra-fraction lesion movement and close organs at risk (OARs) such as the bladder.

The aim of the present radiotherapy planning study was therefore to investigate the dosimetric feasibility of a LARC neoadjuvant radiotherapy protocol administering VMAT-FFF hypofractionated 5-10 Gy boost to restricted diffusion subvolumes (phase 1, 5-10 Gy/1#) prior to conventional long-course VMAT treatment (phase 2, 50 Gy/25#), through comparison to prior treatment plans and standard dosimetry targets.

## Materials and methods

This retrospective study was approved by a local Human Research Ethics Committee.

### Patient selection

The departmental database was retrospectively searched for patients who underwent curative-intent NCRT for low- or mid-rectal T3-T4 tumours who had dedicated pre-treatment CT and MRI from 2018 onwards. Patients without the required pre-treatment imaging, patients who had received inguinal nodal radiotherapy, or who had a history of previous radiotherapy treatment were excluded.

### Imaging

All patients underwent pre-treatment simulation CT (Siemens SOMATOM; 120 kV, in-plane resolution 1.27 mm, slice thickness 2 mm) and MRI (1.5T Siemens MAGNETOM Aera) consisting of axial T2-weighted (repetition time (TR) = 4220 ms, echo time (TE) = 108 ms, in-plane resolution = 1.04 mm, matrix = 384 × 384, slice thickness = 3 mm, slice gap = 0) and diffusion weighted acquisitions (TR = 8100 ms, TE = 61 ms, in-plane resolution = 2.67 mm, matrix = 150 × 132, slice thickness = 3 mm, slices = 38, slice gap = 0, *b* values = [50,400,800]). MR images were rigidly co-registered to the planning CT using in-built tools in MIM Maestro (v6.9.7, MIM Software, Inc., Cleveland, OH), subsequently manually checked by the study radiation oncologists (ROs).

### Contouring

#### Standard treatment volumes

Existing treatment plans (10 MV VMAT) were retrieved from the departmental treatment planning system (TPS; MONACO v5.51, Elekta AB, Stockholm, Sweden). All prior treatment plans were created in line with standard departmental protocol, EviQ^14^ and RTOG guidelines^15^ for conventional NCRT, including standard volumes for planning target volumes (PTV-4500 cGy, PTV-5000 cGy), clinical target volumes (CTV) and OARs (small bowel, bowel cavity, bladder, large bowel, femoral heads).

#### Boost volumes

Previously, a semi-automated approach to delineating tumour subvolumes using co-registered T2-weighted and DWI was described, where visually identified regions of restricted diffusion within a gross tumour volume (*GTV*) delineated by expert observers demonstrated moderate agreement with subvolumes segmented using the ≤40th centile intra-tumoural ADC value.^11^ The same method was used here, with a restricted diffusion subvolume (denoted the *C40* volume) segmented from a GTV contoured by the study ROs using fused T2-weighted and DWI sequences in MIM Maestro, before export to the TPS. After automatic cleaning of non-contiguous voxels and checking by the ROs, the boost target volume (*BTV*) was created by applying margins of 11 mm anteroposteriorly, 7 mm transverse, and 13 mm craniocaudally.^16^ BTV margins were limited to the CTV.

### Treatment planning

Prior treatment plans used 10 MV VMAT with a dual 360° coplanar arc, collimator angle 5°, 0.29 cm calc grid, 180 control points. All new patient plans were retrospectively optimized by the same dosimetrist using the TPS Monte Carlo algorithm. The same patient-specific PTV and OAR contours from the previous treatment plans were used across all re-plans.

#### Phase 1 plans

Phase 1 plans (5, 7, or 10 Gy/1#) used 6 MV VMAT-FFF, with single 360° coplanar arc, collimator angle 5°, 0.20 cm calc grid, 130 control points. The 0.20 cm calculation grid size was chosen based on SBRT guidelines^17^ and control points were limited to minimize excessive beam modulation, considering factors such as intra-fractional motion, MLC leaf interplay, and usual departmental practices.

#### Phase 1 + 2 plans

Combined plans were created with biased-dose planning (phase 1 = 5, 7, or 10 Gy/1# followed by phase 2 = 45-50 Gy/25# SIB). The conventional phase 2 plan used the same standard VMAT parameters as the prior treatment plans.

#### Plan optimization, aims, and acceptance parameters

Dose constraints for phase 1 treatment are summarized in Table 1. Mandatory treatment volume (C40, BTV) dose targets and acceptable variations were based on standard departmental SBRT protocol targets. An evaluation structure (*EVAL*) for dose spill outside the C40 volume was created by subtracting the (C40 + 1mm expansion) volume from the BTV volume. Dose within the resultant structure was limited to <110% of the prescribed boost dose. Minor and major violations were defined as where dose targets could not meet clinically acceptable and mandatory targets, respectively.

**Table 1.** Treatment planning constraints for phase 1 boost.

Structure		Primary constraint	Acceptable variation
Mandatory			
C40 (<40th centile ADC subvolume)	D100%	>100%	–
	D0.03cc	<125%	< 130%
	Dmean	>100%	–
BTV (C40 + expansions)	D95%	>100%	–
	D99%	>90%	–
	Dmax	<120%	<125%
	Dmean	>100%	–
EVAL	V(110% px)	<1%	–
PDS		<1.10	<1.15
MGI		<4.5	<5
Secondary			
R50		PTV dependent	PTV dependent
NTT_2cm		PTV dependent	PTV dependent
NTT		<1 cc	
Bladder dose	Dmean	<35%	–

Abbreviations: ADC, apparent diffusion coefficient; CI, confidence interval; EVAL, dose fall-off evaluation structure; PDS, prescription dose spillage; MGI, modified gradient index; NTT, non-target tissue; and px, prescribed dose.

Conformality of the BTV was also evaluated. The prescription dose spillage (PDS) and modified gradient index (MGI) were utilized as conformality constraints as per the Stereotactic Ablative Therapy Consortium guidelines.^18^ The R50 and NTT maximum dose at 2 cm from the treatment volume (NTT_2cm) were linearly interpolated or extrapolated from RTOG 0813 reference values according to PTV volumes^19^; along with mean bladder dose, these were optimized throughout planning but were not used as strict constraints. The NTT volume receiving prescription dose was also minimized. The RTOG CI^20^ and Paddick CI^21^ were calculated but were not specifically optimized for in planning.

For combined phase 1 + 2 plans, PTV objectives followed ICRU 83 guidelines^22^ and were mandatory. OAR targets followed departmental practices and standard eviQ guidelines for NCRT.^14^ Doses to OARs were considered as optimal where meeting usual targets, or minor or major exceptions depending on the extent by which goals were not met in line with usual clinical assessment. All plans went through an average of 3 optimization iterations with reference to PlanIQ feasibility software (v2.2, Sun Nuclear Corp., Melbourne, FL, USA) to achieve best OAR doses.

#### Quality assurance and deliverability

All plans were independently checked by a second radiation therapist and reviewed by an RO for clinical suitability. Accuracy of TPS planned dose was evaluated by adapting the local SBRT quality assurance (QA) protocol, including measurements with a high-resolution detector array used for SBRT plan validation (Octavius 1600 SRS detector, Octavius 4D rotating cylindrical phantom; PTW, Freiburg, Germany). All treatment plans were delivered on an Elekta Versa HD linear accelerator and compared with calculated doses using the established gamma analysis method^23^ with PTW Verisoft (PTW, Freiburg, Germany). A pass rate of 95% (criteria: 2%/2 mm) or more was required to be accepted in line with QA of single- and hypofractionated treatment. The total beam-on time for each treatment field was measured. Finally, an independent calculation of monitor units (MU) and physics QA was undertaken using DoseCheck (Sun Nuclear Corp.), with a gamma pass rate of >95% (criteria: 3%/3 mm) for the entire calculated volume.

### Statistical evaluation

DVH data were exported from the TPS with 0.5 cGy binning. Mean, median, and interquartile (IQR) range values were calculated for each dose group. Counts of minor/major violations were recorded. Statistical analysis was performed in the R Statistical Environment (v4.3, R Core Team, Vienna, Austria).

## Results

### Patient population

Seventeen patients were initially identified for inclusion. Four were excluded due to previous prostate radiotherapy and 2 for inguinal nodal radiotherapy. One further patient (mid–high tumour location, female) had significant tumour motion between CT and MRI acquisitions with differences in bladder filling and was not suitable for inclusion.

Ten patients were therefore included in the analysis. Patient characteristics are summarized in Table 2. The sample comprised of 6 males and 4 females with a median age (±IQR) of 52.5 (±12). Median GTV was 52.7 cc (±18.5 cc) and tumour length was 5.0 cm (±2.9 cm).

**Table 2.** Patient and tumour characteristics.

Patient	Sex	Tumour staging	Tumour length (cm)	Tumour location	Phase 1 (boost)		Phase 2 (conventional)		
					C40 (cc)	BTV (cc)	GTV (cc)	CTV (cc)	PTV (cc)
1	M	T3N1	6.5	Low	21.5	171.2	55.3	311.2	487.3
2	F	T3N2	6.0	Mid	14.7	106.8	37.5	228.5	374.2
3	M	T3N1	4.8	Low	29.7	185.4	49.6	404.4	707.7
4	M	T4N0	7.0	Low	13.2	96.5	63.8	185.0	308.0
5	M	T3N1	5.0	Mid	34.9	142.1	50.2	297.8	517.7
6	F	T3N1	2.9	Mid	5.2	40.3	11.3	286.2	501.1
7	F	T3N2	5.0	Mid	35.4	138.1	64.7	212.4	341.4
8	M	T3N0	3.0	Low	29.0	98.4	42.5	155.6	380.9
9	M	T3N2	7.0	Mid	44.4	170.3	80.2	409.6	695.4
10	F	T3N2	3.0	Mid	38.1	162.0	59.7	491.8	1046.0

### Phase 1 analysis

**Figure 1.**
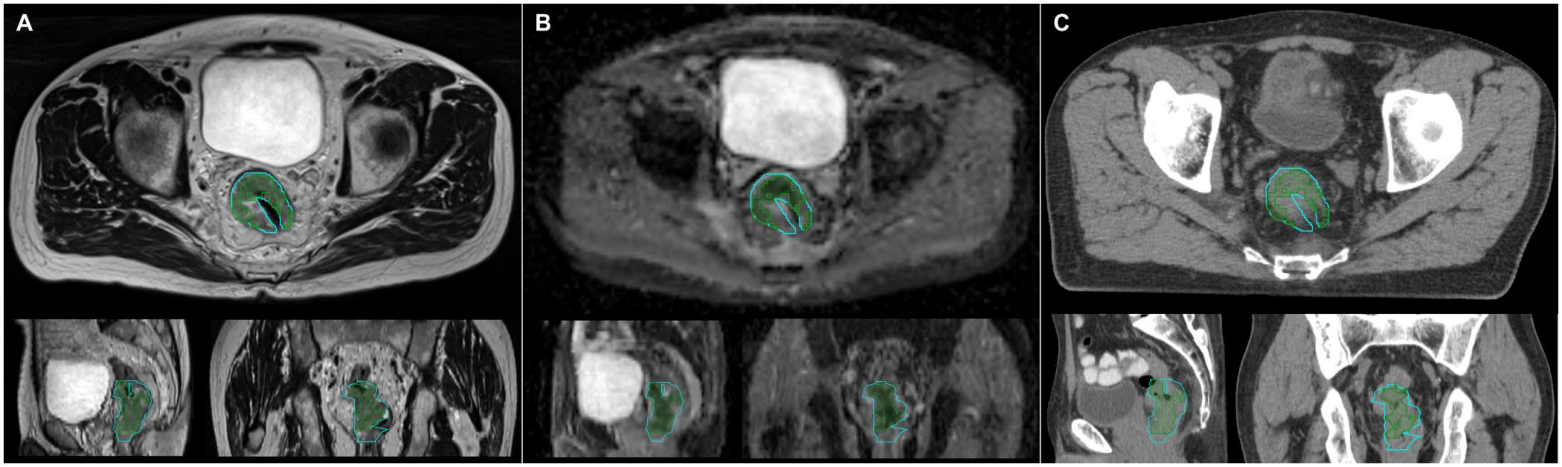
Example of GTV (cyan) and C40 (green) contours on (A) T2, (B) DWI, and (C) CT.

Dosimetry data for the delivery of the upfront boost phase are summarized in Table 3. Contouring was completed with acceptable registration between planning CT and MRI (see Figure 1). All phase 1 plans met QA tolerances (see Supplementary Material, Supplement A).

**Table 3.** Phase 1 boost delivery and conformality evaluation.

		5 Gy		7 Gy		10 Gy	
		Dose	Violations	Dose	Violations	Dose	Violations
Dose delivery							
C40	D0.03 cc (Gy)	5.82 ± 0.39	0	8.26 ± 0.36	0	11.54 ± 0.30	0
	D100% (Gy)	5.06 ± 0.12	2	7.13 ± 0.17	2	10.12 ± 0.15	2
	Dmean (Gy)	5.44 ± 0.12	0	7.64 ± 0.12	0	10.82 ± 0.13	0
BTV	D0.03 cc (Gy)	5.82 ± 0.39	0	8.26 ± 0.36	0	11.54 ± 0.30	0
	D95% (Gy)	5.01 ± 0.01	0	7.01 ± 0.01	0	10.02 ± 0.02	0
	D99% (Gy)	4.91 ± 0.01	0	6.87 ± 0.02	0	9.83 ± 0.05	0
	Dmean (Gy)	5.28 ± 0.04	0	7.38 ± 0.04	0	10.50 ± 0.07	0
EVAL	V(110% px)	0.37 ± 0.22	0	0.14 ± 0.23	0	0.02 ± 0.09	0
MU		1125.78 ± 28.88	–	1503.98 ± 155.98	–	2124.65 ± 168.92	–
Treatment time (s)		168.45 ± 14.12	–	168.14 ± 10.75	–	167.17 ± 6.51	–
Conformality							
PDS		1.03 ± 0.01	0	1.03 ± 0.02	1/0	1.04 ± 0.01	1/0
MGI		3.67 ± 0.07	0	3.72 ± 0.11	1/0	3.87 ± 0.11	1/0
R50		3.5 ± 0.10	7/3	3.55 ± 0.18	7/3	3.8 ± 0.18	6/4
NTT		0.79 ± 0.27	3	0.91 ± 0.42	5	1.16 ± 0.34	8
NTT_2cm		56.8 ± 3.03	0	56.80 ± 3.80	0	59.00 ± 1.70	0
RTOG CI		0.96 ± 0.01	–	0.95 ± 0.01	–	0.96 ± 0.01	–
Paddick CI		0.93 ± 0.01	–	0.93 ± 0.02	–	0.92 ± 0.02	–
Bladder Dmean		1.27 ± 0.55	2	1.92 ± 0.60	1	3.09 ± 0.80	2

Doses expressed as median ± IQR. Violations expressed as minor/major instances where applicable.

Abbreviations: CI, confidence interval; EVAL, dose fall-off evaluation structure; MU, monitor units; PDS, prescription dose spillage; MGI, modified gradient index; NTT, non-target tissue; px, prescribed dose.

#### Deliverability

**Figure 2.**
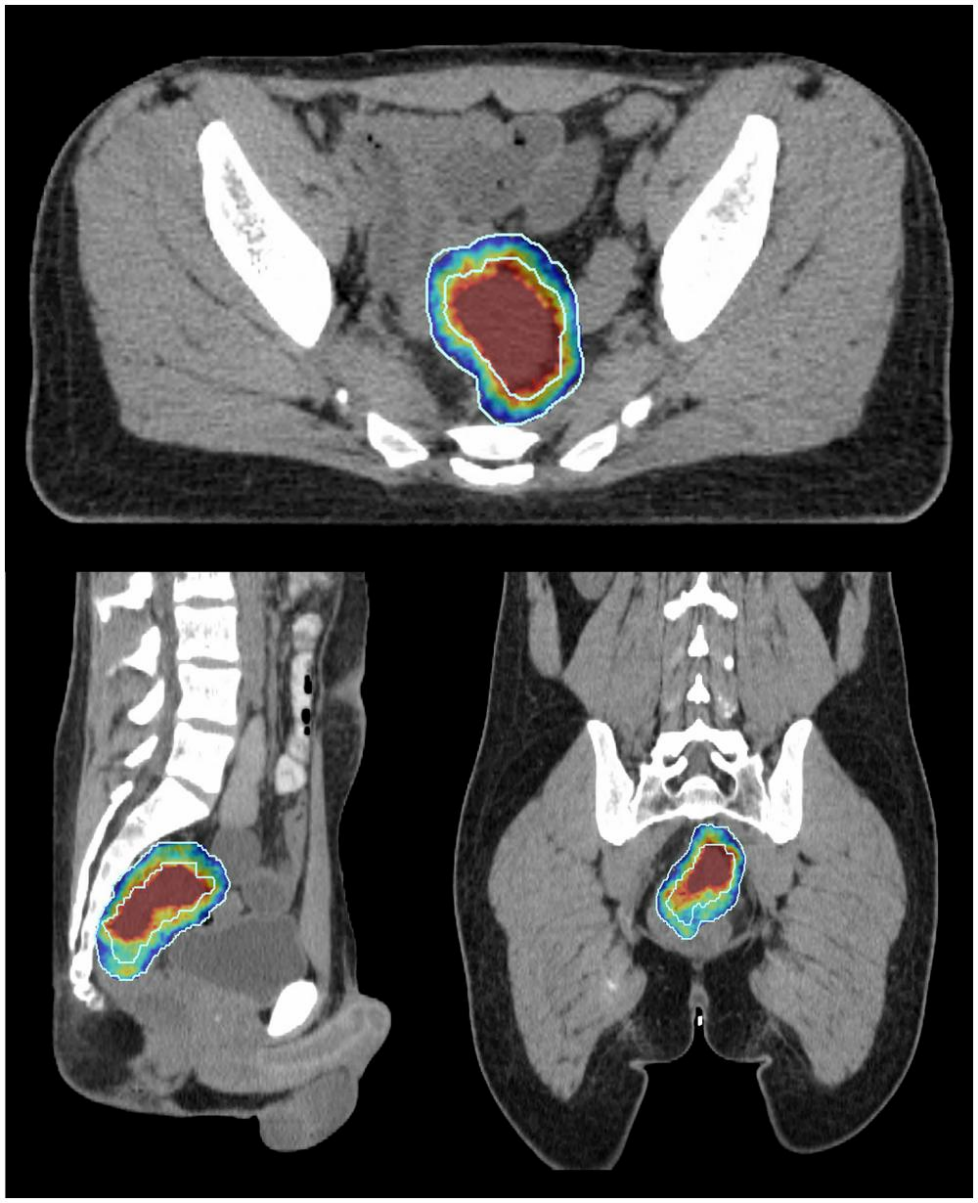
Colourmap of dose distribution within phase 1 boost plan scaled from 100% to 110% of prescription dose. EVAL contour (light blue) used to confirm dose fall-off from central C40 volume.

Dose targets for the BTV were met for all cases at all dose levels. C40 targets were achievable except the D100% in 2 instances at 5 Gy (4.93 and 4.86 Gy), 1 instance at 7 Gy (6.95 Gy), and 2 instances at 10 Gy (9.95 and 9.85 Gy). These exceptions occurred in the same 2 patients, where the inferior margin of the C40 volume was limited by the inferior limit of the CTV. EVAL structure constraints were met in all instances (see Figure 2). Boost MU increased as expected with higher doses; however, delivery time remained similar.

#### Conformality

**Figure 3.**
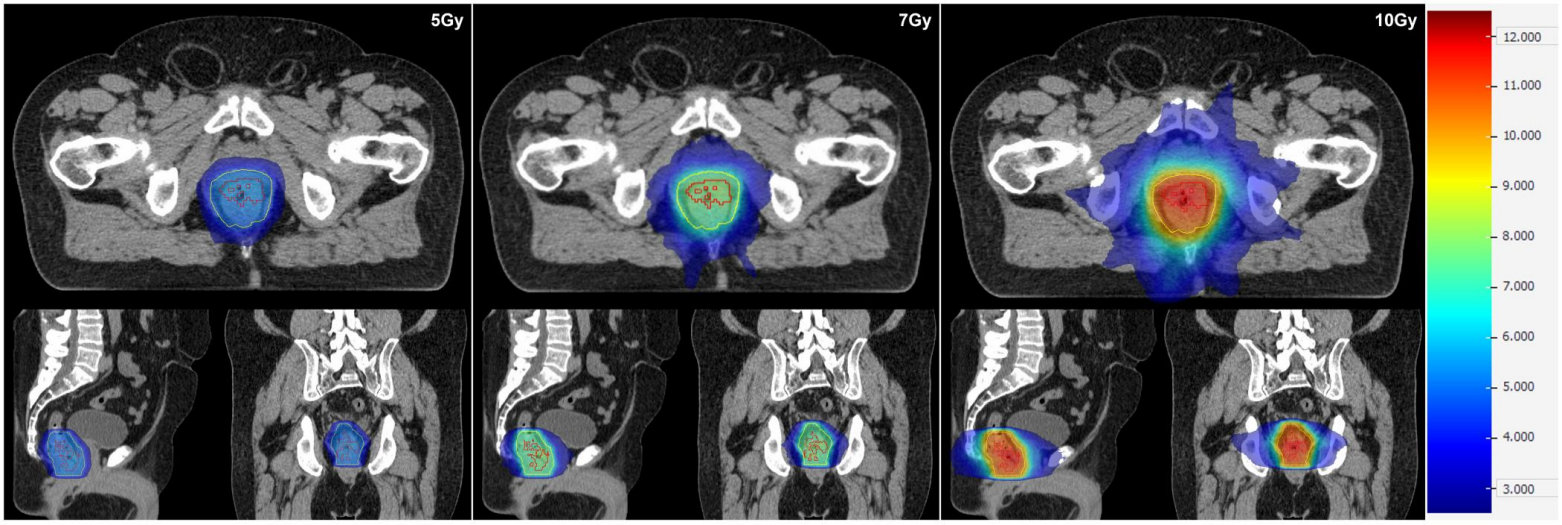
Phase 1 boost plans at 5, 7, and 10 Gy. Standardized dose colourmap across all plans. Yellow contour: BTV; red contour: C40.

PDS and MGI were met in all cases at 5 Gy. One minor violation occurred in 7 Gy (PDS 1.13, MGI 4.88) and 10 Gy (PDS 1.11, MGI 4.87) planning of the same patient who had the smallest treatment volumes (C40 5.2 cc, BTV 40.3 cc). Reference R50 values were not able to be met. NTT_2cm was met in all instances, while the NTT <1 cc constraint was met in 7 cases at 5 Gy, 5 cases at 7 Gy, and 2 cases at 10 Gy. RTOG CI and Paddick CI were acceptable or ideal in all cases at all dose levels. Bladder Dmean was exceeded in 2 instances at 5 Gy (1.90 and 1.55 Gy), 1 instance at 7 Gy (2.63 Gy), and 2 instances at 10 Gy (3.82 and 3.55 Gy). See Figure 3.

### Phase 1 ± 2 analysis

**Figure 4.**
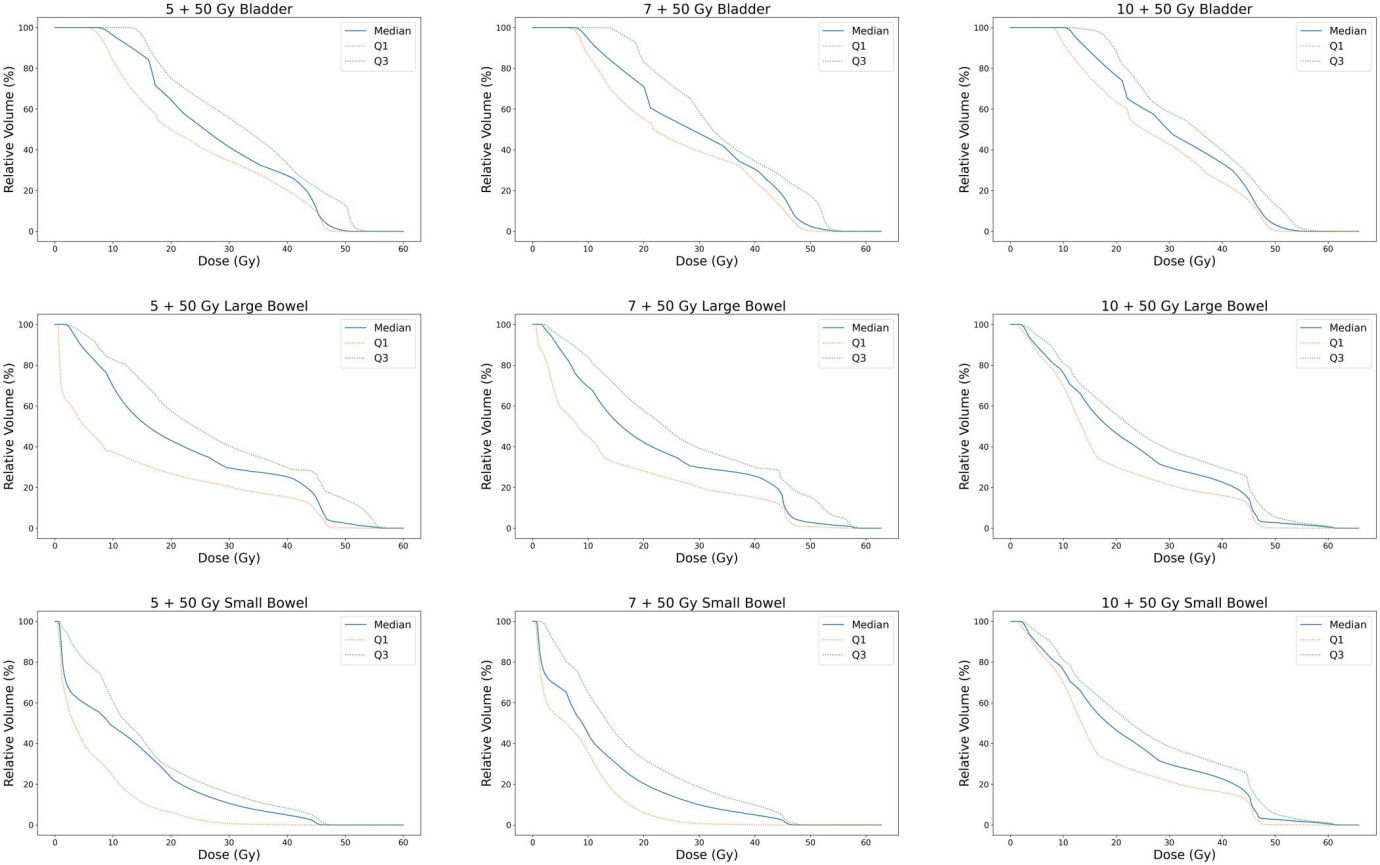
Population dose–volume histograms for combined phase 1 + 2 plans.

Boosted treatment plans were developed with biased-dose planning as detailed (see Table 4 and Figure 4). All plans met usual CTV/PTV targets and QA tolerances (see Supplementary Material, Supplement A). In several instances, dose to relevant OARs was lower than the previously delivered non-boosted plans due to improved planning optimization.

**Table 4.** Medians of target structure and OAR dosimetry of combined phase 1 + 2 biased-dose re-planning, and comparison with previously administered non-boosted plans.

Structure		Existing plan (50 Gy)		5 + 50 Gy		7 + 50 Gy		10 + 50 Gy	
		Dosimetry	Violations	Dosimetry	Violations	Dosimetry	Violations	Dosimetry	Violations
PTV4500 (phase 2 only)	D98%	99.49 ± 0.31	0	98.81 ± 0.64	0	98.52 ± 1.34	0	98.85 ± 1.22	0
	D2%	0.13 ± 0.46	0	0.34 ± 0.21	0	0.44 ± 0.50	0	0.37 ± 0.34	0
	Dmean	45.62 ± 0.52	0	45.87 ± 0.36	0	45.69 ± 0.44	0	45.63 ± 0.47	0
PTV5000 (phase 2 only)	D98%	99.94 ± 0.42	0	99.66 ± 0.83	0	99.73 ± 0.46	0	99.90 ± 0.40	0
	D2%	0.10 ± 0.35	0	0.13 ± 0.19	0	0.05 ± 0.08	0	0.08 ± 0.18	0
	Dmean	50.88 ± 0.50	0	51.00 ± 0.20	0	51.01 ± 0.40	0	51.08 ± 0.18	0
Bladder	V40 (<40%)	28.90 ± 7.90	0/2	30.13 ± 11.13	0/2	30.47 ± 9.85	0/2	33.98 ± 15.67	1/2
	V45 (<15%)	12.82 ± 10.02	1/3	13.13 ± 13.60	0/4	17.57 ± 15.29	1/4	24.09 ± 12.44	1/6
	Dmax (<50 Gy)	48.47 ± 3.14	2/0	51.00 ± 5.61	1/4	52.16 ± 6.05	0/5	53.38 ± 7.31	1/5
Bowel cavity	V45 (<195 cc)	62.08 ± 84.05	0/1	50.16 ± 46.38	1/1	62.46 ± 76.07	1/1	63.42 ± 72.48	1/1
	D1cc (minimize)	47.42 ± 4.48	–	48.09 ± 8.03	–	51.23 ± 9.02	–	49.05 ± 12.76	–
Large bowel	V30 (<200 cc)	104.12 ± 98.88	0/2	99.30 ± 94.83	1/1	97.19 ± 101.64	2/0	102.00 ± 102.42	2/0
	V35 (<150 cc)	84.74 ± 90.57	0/2	83.29 ± 90.40	0/2	82.60 ± 96.23	0/2	86.26 ± 95.68	0/2
	V45 (<20 cc)	35.37 ± 80.41	1/6	43.72 ± 62.93	0/7	44.55 ± 68.48	0/7	61.23 ± 74.06	0/8
Small bowel	V15 (<120 cc)	136.07 ± 119.25	1/5	141.80 ± 112.85	1/5	93.01 ± 124.80	0/4	102.92 ± 132.91	0/4
	D1cc (<50 Gy)	45.57 ± 7.01	1/0	45.61 ± 9.71	1/1	46.45 ± 11.55	0/2	45.85 ± 12.47	0/2

Doses expressed as median ± interquartile range. Violations expressed as minor/major instances where applicable.

Across the median data, the key OARs not meeting standard constraints were the bladder (V45 and Dmax) and large bowel (V45), which tended to increase with increasing boost dose. Dose to other OARs overall remained comparable across boost levels, with most violations occurring in instances where tolerances were borderline or exceeded on the originally delivered non-boosted plans, but had been deemed clinically acceptable to meet PTV targets. Bilateral femoral head/neck doses were well within constraints in all instances.

## Discussion

To the authors’ knowledge this is the first study to evaluate the feasibility of hypofractionated dose escalation to intra-tumoural volumes of restricted diffusion in LARC, building on the prior contouring study.^11^ The key rationale of this approach is to maximize the utility of routinely acquired pre-treatment imaging, while integrating functional information to optimize the therapeutic ratio of local disease control to toxicity. In the absence of adaptive planning such as that provided by MR-LINAC therapy,^24^ which has limited availability internationally, the method proposed here may reduce the risk of boosting NTT, given the significant changes to tumour volume^25^ and diffusion characteristics^26^ that occur throughout NCRT.

While VMAT-FFF is an established method for SBRT and hypofractionated therapy for other tumour sites, there are limited data on its use in rectal radiotherapy. Ding et al.^27^ found similar OAR doses between 6 MV VMAT and 6 MV VMAT-FFF techniques, but higher PTV V105% with the use of VMAT-FFF. The ability to drive higher dose to the target volumes with VMAT-FFF was advantageous for the present protocol, allowing targeted dose escalation to the C40 subvolume while maintaining a steep drop-off within the remainder of the BTV, as validated by the EVAL structure. Except for 2 cases where the margins of the C40 volume were close to the CTV cut-off inferiorly, dosimetry targets for treatment volumes were met for both C40 and PTV at all dose levels. This was achieved while limiting overall plan complexity through the use of single arc therapy and an intermediate number of control points, given considerations of overall treatment time and possible intra-fractional target motion.

Conformality was evaluated with reference to SBRT metrics, given the intended delivery of a highly conformal hypofractionated boost. Aside from 1 minor violation at 5 and 7 Gy planning of the smallest tumour volume in the study population, the MGI and PDS constraints were met by all boost plans. RTOG and Paddick conformity indices were also satisfactory across all plans. R50 constraints could not be met, while NTT_2cm, another measure of intermediate dose spill, was met in all instances. Desai et al.^28^ examined the relationship between PTV geometry and R50 measures, finding strong correlation between increasing R50 values and PTV surface area. At non-lung or -liver tumour sites, or with tumour subvolumes, non-spherical (greater surface area) target volumes are much more likely to be encountered; additionally, given the extrapolation from RTOG 0813 reference values that was required for some PTVs, R50 was not thought to represent a useful evaluation or optimization parameter. A similar geometric effect, along with plan complexity limitations, may also underlie the exceptions in high-dose spillage (NTT) that were observed. Otherwise, VMAT-FFF met the deliverability and conformity requirements.

Target doses to standard PTV and CTV structures were met in all biased-dose phase 1 + 2 plans. Interestingly, many phase 1 + 2 plans, even including boost doses up to 10 Gy, demonstrated lower doses to most OARs than previously delivered clinically acceptable standard NCRT plans, despite unchanged treatment delivery parameters for the phase 2 (conventional VMAT) component. This may have been achieved through departmental protocol optimizations and the implementation of personalized dosimetry optimization software in the interim period. While 5 Gy boost plans showed a minor increase in small bowel V15 compared with prior treatment plans, this was subsequently reduced at 7 and 10 Gy; similarly, the large bowel V30 and V35 were slightly higher in 5 Gy compared with 7 Gy boost plans (see Table 2). While further planning optimization at 5 Gy could likely have reduced these OAR doses, these variances were not dose-limiting.

The main restrictions to escalated dose deliverability in this study were violations in bladder (V45 and Dmax) and large bowel (V45) constraints, which even with biased-dose planning tended to increase across dose levels in the combined plans (see Table 4). As noted, the majority of *a priori* dose-constraint violations occurred where the initially delivered plan also could not meet constraints. With regard to the large bowel, this may be due to adjacency of the treatment field with the OAR structure, as violations predominantly occurred with high-dose spillage (V45). Similarly, dose to the bladder may be least mitigable due to its proximity and potential overlap with the BTV and PTV. Thus, while recent dose escalation studies have generally found comparable acute toxicity between boosted and non-boosted treatment arms,^6^^,^^29^ appropriate patient selection is necessary as underlying anatomical challenges may have significant impact on maximal achievable target dose or PTV coverage.

Additionally, set-up reproducibility including patient bladder and bowel preparation requires optimization for both treatment delivery and planning with co-registered imaging modalities. Cases in the current analysis used only standard pre-treatment fluid loading protocols. One mid- to high-rectal case otherwise meeting inclusion criteria was not useable due to tumour movement between planning CT and MRI, largely due to inconsistent bladder filling. Potential errors in restricted diffusion subvolume delineation due to bowel luminal content variation were also previously noted.^11^ Additional measures such as the use of night-before bowel preparation, anti-peristaltic agents, and confirmation of bladder volume with ultrasound prior to imaging or treatment may reduce variability.^30^

The strengths of this study include a robust end-to-end assessment of the proposed methodology, including semi-automated subvolume delineation, dose optimization, and patient-specific QA. The manuscript scores 96% when assessed with the RATING tool^31^ (see Supplementary Material, Supplement B). Generalization of the findings is limited by the small sample size. The median tumour size in the present study was 52.7 cc and the population was limited to low- and mid-rectal tumours. Mobile tumours, particularly of the upper rectum, or smaller treatment volumes may be challenging for SBRT-style dose-escalated therapies and would require reproducible patient preparation and strict QA to be feasible. Further assessment in a broader cohort with respect to bowel and bladder set-up optimization and validation with on-line cone-beam CT is required.

Other related avenues for research include improving on-line imaging to facilitate more concise PTV expansions, such as with implanted fiducial markers^32^ or MR-LINAC therapy. Combining DWI with other functional MRI series may also improve semi-automated segmentation methods, as described by Knuth et al.^33^ While long-course NCRT remains standard of care at our institution, outcomes have been similar in recent short-course trials.^1^^,^^34^ Devlin et al.^35^ evaluated the dosimetric feasibility of dose-escalated short-course therapy up to 35 Gy/5#; whether DWI-guided escalation could be applied in this context as either upfront or integrated boost could also be investigated.

## Conclusion

In summary, dose-escalated radiotherapy including 5-10 Gy boost to restricted diffusion tumour subvolumes in LARC appears to be feasible with the use of highly conformal VMAT-FFF. Usual OAR dose constraints are generally achievable; however, maximal dose may be dependent on underlying patient anatomical factors and optimization of bowel/bladder preparation. A prospective feasibility trial is currently planned (ACTRN: 12620000757910).

## Supplementary Material

tzad001_Supplementary_Data

## References

[1] Ngan SY , Burmeister B, Fisher RJ, et al Randomized trial of short-course radiotherapy versus long-course chemoradiation comparing rates of local recurrence in patients with T3 rectal cancer: Trans-Tasman Radiation Oncology Group Trial 01.04. J Clin Oncol. 2012;30(31):3827-3833. 23008301 10.1200/JCO.2012.42.9597

[2] Habr-Gama A , Perez RO, Nadalin W, et al Operative versus nonoperative treatment for stage 0 distal rectal cancer following chemoradiation therapy: long-term results. Ann Surg. 2004;240(4):711-717; discussion 7–8. 15383798 10.1097/01.sla.0000141194.27992.32 PMC1356472

[3] Burbach JP , den Harder AM, Intven M, van Vulpen M, Verkooijen HM, Reerink O. Impact of radiotherapy boost on pathological complete response in patients with locally advanced rectal cancer: a systematic review and meta-analysis. Radiother Oncol. 2014;113(1):1-9. 25281582 10.1016/j.radonc.2014.08.035

[4] Hearn N , Atwell D, Cahill K, et al Neoadjuvant radiotherapy dose escalation in locally advanced rectal cancer: a systematic review and meta-analysis of modern treatment approaches and outcomes. Clin Oncol (R Coll Radiol). 2021;33(1):e1-e14. 32669228 10.1016/j.clon.2020.06.008

[5] Appelt AL , Pløen J, Vogelius IR, Bentzen SM, Jakobsen A. Radiation dose–response model for locally advanced rectal cancer after pre-operative chemoradiotherapy. Int J Radiat Oncol Biol Phys. 2013;85(1):74-80. 22763027 10.1016/j.ijrobp.2012.05.017 PMC3539741

[6] Couwenberg AM , Burbach JPM, Berbee M, et al Efficacy of dose-escalated chemoradiation on complete tumor response in patients with locally advanced rectal cancer (RECTAL-BOOST): a phase 2 randomized controlled trial. Int J Radiat Oncol Biol Phys. 2020;108(4):1008-1018. 32565319 10.1016/j.ijrobp.2020.06.013

[7] Verweij ME , Hoendervangers S, Couwenberg AM, et al Impact of dose-escalated chemoradiation on quality of life in patients with locally advanced rectal cancer: 2-year follow-up of the randomized RECTAL-BOOST trial. Int J Radiat Oncol Biol Phys. 2022;112(3):694-703. 34634436 10.1016/j.ijrobp.2021.09.052

[8] van der Heide UA , Houweling AC, Groenendaal G, Beets-Tan RGH, Lambin P. Functional MRI for radiotherapy dose painting. Magn Reson Imaging. 2012;30(9):1216-1223. 22770686 10.1016/j.mri.2012.04.010 PMC5134673

[9] Zamboglou C , Thomann B, Koubar K, et al Focal dose escalation for prostate cancer using (68)Ga-HBED-CC PSMA PET/CT and MRI: a planning study based on histology reference. Radiat Oncol. 2018;13(1):81. 29716617 10.1186/s13014-018-1036-8 PMC5930745

[10] Alongi F , Fersino S, Mazzola R, et al Radiation dose intensification in pre-operative chemo-radiotherapy for locally advanced rectal cancer. Clin Transl Oncol. 2017;19(2):189-196. 27271749 10.1007/s12094-016-1522-0

[11] Hearn N , Bugg W, Chan A, et al Manual and semi-automated delineation of locally advanced rectal cancer subvolumes with diffusion-weighted MRI. Br J Radiol. 2020;93(1114):20200543. 32877210 10.1259/bjr.20200543 PMC7548370

[12] Rosa C , Gasparini L, Di Guglielmo FC, et al DWI-MR and PET-CT functional imaging for boost tumor volume delineation in neoadjuvant rectal cancer treatment. In Vivo. 2023;37(1):424-432. 36593016 10.21873/invivo.13095 PMC9843791

[13] Xiao Y , Kry SF, Popple R, et al Flattening filter-free accelerators: a report from the AAPM Therapy Emerging Technology Assessment Work Group. J Appl Clin Med Phys. 2015;16(3):5219. 26103482 10.1120/jacmp.v16i3.5219 PMC5690108

[14] Cancer Institute NSW. Rectal neoadjuvant EBRT Chemoradiation Pre-operative Long-Course (v.6). 2022. https://www.eviq.org.au/radiation-oncology/colorectal/1863-rectal-neoadjuvant-ebrt-chemoradiation-pre-op. Accessed November 4, 2023.

[15] Gay HA , Barthold HJ, O’Meara E, et al Pelvic normal tissue contouring guidelines for radiation therapy: a radiation therapy oncology group consensus panel atlas. Int J Radiat Oncol Biol Phys. 2012;83(3):e353-e62. 22483697 10.1016/j.ijrobp.2012.01.023 PMC3904368

[16] Kleijnen JJE , van Asselen B, Van den Begin R, et al MRI-based tumor inter-fraction motion statistics for rectal cancer boost radiotherapy. Acta Oncol. 2019;58(2):232-236. 30444161 10.1080/0284186X.2018.1532598

[17] Foote M , Bailey M, Smith L, et al Guidelines for safe practice of stereotactic body (ablative) radiation therapy. J Med Imaging Radiat Oncol. 2015;59(5):646-653. 26122017 10.1111/1754-9485.12336

[18] SABR Consortium. Stereotactic Ablative Body Radiotherapy (SBRT): A Resource. 2019. https://www.sabr.org.uk/wp-content/uploads/2019/04/SABRconsortium-guidelines-2019-v6.1.0.pdf. Accessed November 4, 2023.

[19] Desai DD , Johnson EL, Cordrey IL. An analytical expression for R50% dependent on PTV surface area and volume: a lung SBRT comparison. J Appl Clin Med Phys. 2020;21(11):278-282. 10.1002/acm2.13026 PMC7700934 32996668

[20] Cao T , Dai Z, Ding Z, Li W, Quan H. Analysis of different evaluation indexes for prostate stereotactic body radiation therapy plans: conformity index, homogeneity index and gradient index. Precis Radiat Oncol. 2019;3(3):72-79.

[21] Simon M , Papp J, Csiki E, Kovács Á. Plan quality assessment of fractionated stereotactic radiotherapy treatment plans in patients with brain metastases. Front Oncol. 2022;12:846609. 35345445 10.3389/fonc.2022.846609 PMC8957100

[22] International Commission on Radiation Units and Measurements (ICRU). Prescribing, recording, and reporting photon beam intensity modulated radiotherapy (IMRT). ICRU Report 83. J ICRU. 2010;10(1):1-106.

[23] Low DA , Harms WB, Mutic S, Purdy JA. A technique for the quantitative evaluation of dose distributions. Med Phys. 1998;25(5):656-661. 9608475 10.1118/1.598248

[24] Bonomo P , Lo Russo M, Nachbar M, et al 1.5 T MR-Linac planning study to compare two different strategies of rectal boost irradiation. Clin Transl Radiat Oncol. 2021;26:86-91. 33336086 10.1016/j.ctro.2020.11.016 PMC7732969

[25] Van den Begin R , Kleijnen J-P, Engels B, et al Tumor volume regression during preoperative chemoradiotherapy for rectal cancer: a prospective observational study with weekly MRI. Acta Oncol. 2018;57(6):723-727. 29157069 10.1080/0284186X.2017.1400689

[26] Cai G , Xu Y, Zhu J, Gu W-L, Zhang S, Ma X-J, et al Diffusion-weighted magnetic resonance imaging for predicting the response of rectal cancer to neoadjuvant concurrent chemoradiation. World J Gastroenterol. 2013;19(33):5520-5527. 24023496 10.3748/wjg.v19.i33.5520 PMC3761106

[27] Ding Z , Xiang X, Kang K, Zeng Q, Yuan Q, Xu M. Comparison of dosimetric characteristics between flattening filter-free and flattening filter mode volumetric-modulated arc therapy plans in rectal cancer. Precis Radiat Oncol. 2021;5(2):100-105.

[28] Desai DD , Cordrey IL, Johnson EL. A physically meaningful relationship between R50% and PTV surface area in lung SBRT. J Appl Clin Med Phys. 2020;21(9):47-56. 10.1002/acm2.12964 PMC7497922 32725674

[29] Valentini V , Gambacorta MA, Cellini F, et al The INTERACT Trial: long-term results of a randomised trial on preoperative capecitabine-based radiochemotherapy intensified by concomitant boost or oxaliplatin, for cT2 (distal)–cT3 rectal cancer. Radiother Oncol. 2019;134:110-118. 31005204 10.1016/j.radonc.2018.11.023

[30] Yoon HI , Chung Y, Chang JS, Lee JY, Park SJ, Koom WS. Evaluating variations of bladder volume using an ultrasound scanner in rectal cancer patients during chemoradiation: is protocol-based full bladder maintenance using a bladder scanner useful to maintain the bladder volume? PLoS One. 2015;10(6):e0128791. 26039198 10.1371/journal.pone.0128791 PMC4454439

[31] Hansen CR , Crijns W, Hussein M, et al Radiotherapy Treatment plannINg study Guidelines (RATING): a framework for setting up and reporting on scientific treatment planning studies. Radiother Oncol. 2020;153:67-78. 32976873 10.1016/j.radonc.2020.09.033

[32] van den Ende RPJ , Rigter LS, Kerkhof EM, et al MRI visibility of gold fiducial markers for image-guided radiotherapy of rectal cancer. Radiother Oncol. 2019;132:93-99. 30825976 10.1016/j.radonc.2018.11.016

[33] Knuth F , Groendahl AR, Winter RM, et al Semi-automatic tumor segmentation of rectal cancer based on functional magnetic resonance imaging. Phys Imaging Radiat Oncol. 2022;22:77-84. 35602548 10.1016/j.phro.2022.05.001 PMC9114680

[34] Erlandsson J , Holm T, Pettersson D, et al Optimal fractionation of preoperative radiotherapy and timing to surgery for rectal cancer (Stockholm III): a multicentre, randomised, non-blinded, phase 3, non-inferiority trial. Lancet Oncol. 2017;18(3):336-346. 28190762 10.1016/S1470-2045(17)30086-4

[35] Devlin L , Grocutt L, Hunter B, et al The in-silico feasibility of dose escalated, hypofractionated radiotherapy for rectal cancer. Clin Transl Radiat Oncol. 2022;36:24-30. 35756193 10.1016/j.ctro.2022.06.003 PMC9218294

