# Supplementary material for: Radiotherapy dose escalation using pre-treatment diffusion-weighted imaging in locally advanced rectal cancer: a planning study
Source: BJR Open. 2023 Dec 12;6(1):tzad001. doi: 10.1093/bjro/tzad001 (PMC10860507; doi:10.1093/bjro/tzad001)
Supplement: tzad001_Supplementary_Data [file tzad001_supplementary_data.zip › Supplement A - physics QA.docx]

**Supplement A:**

Physics quality assessment, gamma analysis pass rate by patient and dose level.
